# Genotype–phenotype correlations and nephroprotective effects of RAAS inhibition in patients with autosomal recessive Alport syndrome

**DOI:** 10.1007/s00467-021-05040-9

**Published:** 2021-03-27

**Authors:** Yanqin Zhang, Jan Böckhaus, Fang Wang, Suxia Wang, Diana Rubel, Oliver Gross, Jie Ding

**Affiliations:** 1 Department of Pediatrics, Peking University First Hospital, No. 1 Xi An Men Da Jie, Beijing, 100034 China; 2 Clinic of Nephrology and Rheumatology, University Medical Center Goettingen, Robert-Koch Str. 40, 37075 Goettingen, Germany; 3 Department of Electron Microscopy, Peking University First Hospital, Beijing, 100034 China

**Keywords:** Alport syndrome, Autosomal recessive inheritance, *COL4A3* gene, *COL4A4* gene, Nephroprotection

## Abstract

**Background:**

Autosomal recessive Alport syndrome (ARAS) is caused by pathogenic variants in both alleles of either *COL4A3* or *COL4A4* genes. Reports on ARAS are rare due to small patient numbers and there are no reports on renin-angiotensin-aldosterone system (RAAS) inhibition therapy in ARAS.

**Methods:**

Retrospective study in 101 patients with ARAS from Chinese Registry Database of Hereditary Kidney Diseases and European Alport Registry. Genotype–phenotype correlations and nephroprotective effects of RAAS inhibition in ARAS were evaluated.

**Results:**

Median age was 15 years (range 1.5–46 years). Twelve patients progressed to stage 5 chronic kidney disease (CKD5) at median age 20.5 years. Patients without missense variants had both higher prevalence and earlier onset age of hearing loss, nephrotic-range proteinuria, more rapid decline of eGFR, and earlier onset age of CKD5 compared to patients with 1 or 2 missense variants. Most patients (79/101, 78%) currently are treated with RAAS inhibitors; median age at therapy initiation was 10 years and mean duration 6.5 ± 6.0 years. Median age at CKD5 for untreated patients was 24 years. RAAS inhibition therapy delayed CKD5 onset in those with impaired kidney function (T-III) to median age 35 years, but is undefined in treated patients with proteinuria (T-II) due to low number of events. No treated patients with microalbuminuria (T-I) progressed to CKD5. ARAS patients with 1 or 2 missense variants showed better response to treatment than patients with non-missense-variants.

**Conclusions:**

Our study provides the first evidence for early use of RAAS inhibition therapy in patients with ARAS. Furthermore, genotype in ARAS correlates with response to therapy in favor of missense variants.

## Introduction

Alport syndrome is a hereditary glomerular disease characterized by hematuria, proteinuria, and progressive kidney failure, usually accompanied with sensorineural hearing loss and ocular abnormalities [1–4]. To date, about 85% of patients are diagnosed with X-linked Alport syndrome (XLAS) caused by pathogenic variants in the *COL4A5* gene [5, 6]. The remaining 15% of patients with Alport syndrome, in descending order of the number of cases reported, include autosomal recessive Alport syndrome (ARAS) caused by pathogenic variants in both alleles of either *COL4A3* or *COL4A4* genes [7–9], autosomal dominant Alport syndrome (ADAS) caused by heterozygous pathogenic variants in *COL4A3* or *COL4A4* genes [10, 11] and digenic Alport syndrome caused by coexisting pathogenic variants in *COL4A3*, *COL4A4*, or *COL4A5* genes [12, 13].

Up to now, most reports on Alport syndrome describe patients with XLAS: age at stage 5 chronic kidney disease (CKD 5) strongly correlates with *COL4A5* genotype in males with XLAS [14–16]. However, only a few reports describe patients with ARAS: a systematic review in 2019 included only 26 articles with 148 patients [17]. Furthermore, inhibition of the renin-angiotensin-aldosterone system (RAAS) by angiotensin-converting enzyme inhibitors (ACEi) or angiotensin receptor blockade (ARB) can delay kidney failure in both animal models and human patients with Alport syndrome [18–21]. Until now, no data has been reported on the effects of RAAS inhibition therapy in patients with ARAS. Therefore, this lack of sufficient data on therapeutic options in patients with ARAS led us to consider combining different international databases to enable researchers to analyze a large number of ARAS patients with long-term clinical course [22].

In this study, we describe the clinical and genetic characteristics in 101 patients with ARAS originating from the Chinese Registry Database of Hereditary Kidney Diseases and the European Alport Registry.

## Methods

### Patients

Patients with ARAS were selected from the Chinese Registry Database of Hereditary Kidney Diseases and the European Alport Registry. ARAS was diagnosed on either or both of the following two criteria, (i) kidney biopsy in young patients with typical changes of Alport syndrome, like splitting or lamellation in glomerular basement membrane (GBM), and both parents with hematuria or thin basement membrane nephropathy (TBMN) without proteinuria and CKD5; (ii) homozygous or compound heterozygous mutations (pathogenic variants) in *COL4A3* or *COL4A4* genes. Patients suspected of ARAS who had neither kidney biopsy nor genetic diagnosis, patients who had a heterozygous pathogenic variant in *COL4A3* or *COL4A4* genes, and patients who had coexisting variants in *COL4A3* and *COL4A4* genes were excluded.

Clinical data were retrospectively analyzed including gender, onset age of hematuria, proteinuria, estimated glomerular filtration rate (eGFR) decline and extra-renal manifestations, pathology of kidney biopsy and the age at kidney biopsy, results of genetic test, age at onset of CKD5, and RAAS inhibition therapy. Patients were categorized depending on number of missense variants and kidney function at initiation of RAAS inhibition therapy. Initiation of therapy was defined as follows:

- T-0 starts at patients with microhematuria only
- T-I starts at patients with microalbuminuria
- T-II starts at patients with proteinuria and eGFR ≥60 ml/min × 1.73m^2^
- T-III starts at patients with eGFR <60 ml/min × 1.73m^2^

The study was approved by the Ethical Committee of Peking University First Hospital (2016 [1179] V3.0), informed consent was obtained from the probands and their family members. The European Alport registry was approved by the Ethical Committee of the University Medical Center Goettingen (10/11/06).

### Detection and definition of pathogenic variants

Genomic DNA samples from probands were tested for pathogenic variants by direct sequencing using the Sanger method for all exons and exon–intron boundaries of *COL4A3* and *COL4A4* genes (before 2014) and tested for all three Alport genes by targeted next-generation sequencing (NGS) or exome sequencing (since 2014). Sanger sequencing or qPCR (real-time quantitative PCR) was used to confirm the candidate pathogenic variants in probands and their family members. The pathogenicity of variants was assessed based on the ACMG classification and the expert consensus guidelines for the genetic diagnosis of Alport syndrome [23, 24]. Of note, rarely, some glycine substitutions (p.Gly43Arg in *COL4A3*, p.Gly999Glu in *COL4A4*, and p.Gly545Ala in *COL4A4*) are not pathogenic [25].

### Statistical analysis

Statistical analysis was performed using GraphPad Prism V8 (GraphPad Software, Inc.). Medians and ranges were used for continuous variables. Frequencies and percentages were used for categorical variables. Mann–Whitney test was used to compare skewed distributed variables between two groups. Fisher’s test was used to compare the categorical variables in different groups. Log-rank (Mantel–Cox) test was used for censored time-to-event data. If the *p* value <0.05, the differences were considered to be statistically significant.

## Results

### Clinical features

A total of 101 patients (68 from China, 33 from Europe) with ARAS were enrolled in this study. The general characteristics and clinical features of the patients are shown in Table 1. Twenty-six patients (26/64, 41%) had a family history positive for kidney disease. Only two patients were from consanguineous families (2/68, 3%). The percentage of homozygous variants in ARAS patients was 16% (13/79), which implies heterozygous carriers of *COL4A3* or *COL4A4* variants are common in the population. Thirteen patients (13/46, 28%) had ocular abnormalities, seven of whom had temporal retinal thinning, five perimacular dot-and-fleck retinopathy, and one presented with a cataract. Twenty-five patients (25/51, 49%) had hearing loss (≥ 25 dB HL) and transmission hypoacusis was ruled out by an otolaryngologist with normal otolaryngological physical examination and pure tone audiometry. Twelve patients (12/101, 12%) developed CKD 5 at a median age of 20.5 years (range, 12 to 46 years).

**Table 1:** Patients’ characteristics

Characteristics	Number of patients (%)	Median age in years (range)
All patients	101	15.0 (1.5–46.0)
Male	60/101 (60%)	
Female	41/101 (41%)	
Age < 18	58/101 (58%)	
Age ≥ 18	43/101 (43%)	
Hematuria	68/68 (100%)	4.0 (0.3–32.0)
Proteinuria	57/66 (86%)	7.0 (0.6–33.0)
Nephrotic-range proteinuria	22/57 (39%)	11.0 (4.0–33.0)
Patients with molecular genetic diagnosis*	79/101 (79%)	
*COL4A3* gene	58/79 (73%)	
*COL4A4* gene	21/79 (27%)	
Homozygous	13/79 (16%)	
Compound heterozygous	66/79 (84%)	
Kidney biopsy	66/101 (66%)	8.0 (1.5–33.0)
Patients with hearing loss	25/51 (49%)	10.0 (0.1–33.0)
Patients with ocular lesions	13/46 (28%)	11.0 (4.0–25.0)
Patients with therapy	79/101 (79%)	10.0 (1.0–33.0)
T-0, hematuria	0	
T-I, microalbuminuria	9/79 (11%)	
T-II, proteinuria, eGFR >60	65/79 (83%)	
T-III, eGFR <60	5/79 (6%)	
Patients with CKD 5	12/101 (12%)	20.5 (12.0–46.0)

*Genetic diagnosis of autosomal recessive Alport syndrome in the remaining 22/101 patients was based on the genealogic tree with two parents and other relatives with hematuria (and thin basement membrane disease)

**Fig. 1:**
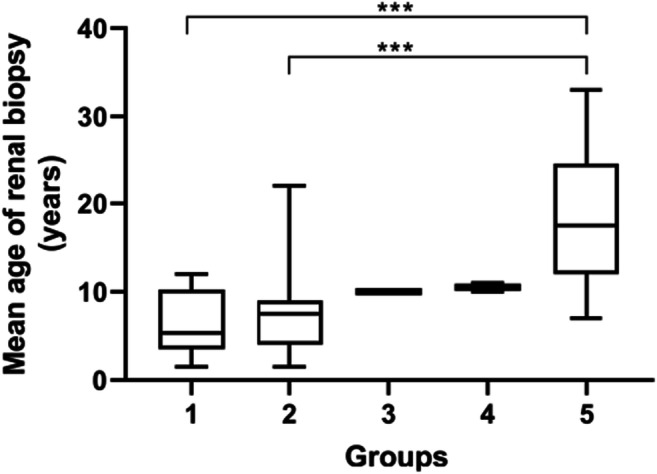
Kidney pathology results by light microscopy in 44 patients^#^ with ARAS. Group 1, Minor glomerular abnormalities (n = 10); group 2, Mesangial proliferative glomerulonephritis (MsPGN, n = 23); group 3, Membranous nephritis (MN, n = 1); group 4, Mesangial proliferative glomerulonephritis and IgA nephropathy (n = 2); group 5, Focal segmental glomerulosclerosis (FSGS, n = 10). ^#^repeat kidney biopsy was performed in 2 patients. ***p < 0.001

Of 66 patients with kidney biopsy, 40 had an electron microscopy examination done with pathological changes like irregular thinning and thickening, splitting, and lamellation in GBM. In 44 patients with reports available for light microscopy, kidney pathology showed very different pathological changes. In children 10 years and younger, the histology changes included mesangial proliferative glomerulonephritis (63%), minor glomerular abnormalities (27%), and others (10%). In contrast, in patients older than 10 years, focal segmental glomerulosclerosis (FSGS) became the typical and most prominent finding (56%). Ten patients (10/44, 23%) showed FSGS by light microscopy and more than 90% of patients with FSGS were older than 10 years. The changes correlated with the age at kidney biopsy, as FSGS was the most common finding in the older patients (Fig. 1). The median age at kidney biopsy in the FSGS group was significantly older compared to the ages in groups of minor glomerular abnormalities and mesangial proliferative glomerulonephritis (17.5 vs. 5.4, *p* < 0.001; 17.5 vs. 7.5, *p* < 0.001; respectively).

### Genetic features

In total, 79 patients (79/101, 79%) had the diagnosis confirmed genetically with pathogenic variants in the *COL4A3* or *COL4A4* genes. Among them, 58 cases (58/79, 73%) were caused by pathogenic variants in the *COL4A3* gene and 21 (21/79, 27%) were caused by pathogenic variants in the *COL4A4* gene. Sixteen percent of them (13/79) were homozygous and 84% (66/79) were compound heterozygous. The identified variants were confirmed to be inherited in trans in parents of 59 patients (59/79, 75%).

### *COL4A3* variants

A total of 58 variants were identified in the *COL4A3* gene, 41 (41/58, 71%) of them were novel (Table 2). There were 24 missense variants (24/58, 41%), 14 frameshift variants (14/58, 24%), nine nonsense variants (9/58, 16%), seven splicing variants (7/58, 12%), three in-frame deletion variants (3/58, 5%), and one variant of initiation codon (1/58, 2%). Of the missense variants, 21 (21/24, 88%) were glycine substitutions in the collagen domain and the other three were missense variants located in the non-collagenous (NC) domain of α3 (IV) chain. Among the glycine substitutions, there were seven replaced by glutamic acid, six replaced by arginine, five replaced by aspartic acid, two replaced by valine, and one replaced by serine. The p.(Leu14_Leu21del) variant was the most frequent *COL4A3* variant, which was detected in nine patients (1 homozygous, 8 compound heterozygous). In addition, the p.(Leu1598Arg) variant was detected in five patients in combination with glycine missense mutations and in one patient in combination with a frameshift variant.

**Table 2:** Characteristics of *COL4A3* variants identified in this study

Exon/intron	Nucleotide change	Amino acid change	Variation type	dbSNP reference ID	ClinVar	ACMG criteria	ACMG	Patient no./zygosity	Reference
1	c.3G>A	p.0?	Init-loss	-	-	PVS1 PM2 PM3 PP3	P	56/ch	Novel
1	c.40_63delCTGCCGCTCCTGCTGGTGCTCCTG	p.Leu14_Leu21del	In-frame deletion	rs774798108	P​	PS4 PM3 PM4 PP1 PP4	P	38/ch; 59/ch; 60/ch; 63/H; 65/ch; 66/ch; 68/ch; 72/ch; 85/ch	[26]
1	c.52_63del	p.Leu18_Leu21del	In-frame deletion	-	-	PS4 PM3 PM4 PP1 PP4	P	72/ch; 95/ch; 86/ch	Novel
3	c.171_172insC	p.Gly58Argfs*12	Frameshift	-	-	PVS1 PM2 PM3 PP3	P	95/ch	Novel
4	c.278_279insGgttagtagtcca	p.Gly94Valfs*40	Frameshift	-	-	PVS1 PM2 PM3 PP3	P	86/ch	Novel
Intron 4	c.279+6T>C	-	Splicing	-	-	PM2 PM3 PP3 PP4	LP	68/ch	Novel
9	c.469G>C	p.Gly157Arg	Missense	rs764451365	VUS	PM2 PM3 PP3 PP4	LP	36/ch	[27]
9	c.522_523insG	p.Leu175Valfs*38	Frameshift	rs761358728	-	PVS1 PM2 PM3 PP3	P	44/ch	Novel
Intron 11	c.645+2t>c	-	Splicing	rs1553752199	LP​	PVS1 PM2 PM3 PP3	P	98/ch; 43/ch	[8]
12	c.679G>C	p.Gly227Arg	Missense	-	-	PM2 PM3 PP3 PP4	LP	78/ch	Novel
13	c.706G>C	p.Gly236Arg	Missense	-	-	PM2 PM3 PP3 PP4	LP	49/ch	Novel
13	c.724_725delG	p.Gly242Glufs*5	Frameshift	-	-	PVS1 PM2 PP3	P	73/H	Novel
15	c.833dupT	p.Pro279Alafs*8	Frameshift	rs1363680371	LP​	PVS1 PM2 PM3 PP3	P	82/H; 93/ch	Novel
16	c.908G>A	p.Gly303Asp	Missense	-	-	PM2 PM3 PP3 PP4	LP	48/ch	Novel
19	c.1038T>A	p.Tyr346*	Nonsense	-	-	PVS1 PM2 PM3 PP3	P	55/ch	Novel
20	c.1133G>T	p.Gly378Val	Missense	-	-	PM2 PM3 PP3 PP4	LP	91/ch	Novel
21	c.1202G>A	p.Gly401Glu	Missense	-	-	PM2 PM3 PP3 PP4	LP	38/ch	Novel
21	c.1216C>T	p.Arg406*	Nonsense	rs371334239	P	PVS1 PM2 PM3 PP3	P	37/H; 65/ch; 60/H; 61/H	[28]
22	c.1323_1324insA	p.Vel442Serfs*25	Frameshift	-	-	PVS1 PM2 PP3	P	99/ch	[8]
22	c.1367_1369delATC	p.Tyr456del	In-frame deletion	rs762420854	VUS	PM2 PM4 PP3 PP4	LP	43/ch	Novel
23	c.1445_1446insAT	p.Pro483Serfs*16	Frameshift	-	-	PVS1 PM2 PP3	P	56/ch	Novel
23	c.1496G>A	p.Gly499Glu	Missense	-	-	PM2 PM3 PP3 PP4	LP	45/ch	Novel
26	c.1908delC	p.Gly637Aspfs*110	Frameshift	-	-	PVS1 PM2 PP3	P	69/ch	Novel
26	c.1927G>A	p.Gly643Ser	Missense	rs778034451	LP​	PM2 PM3 PP3 PP4	LP	64/ch	[29]
Intron 26	c.1928-2A>T	-	Splicing	-	-	PVS1 PM2 PM3 PP3	P	94/ch	Novel
Intron 27	c.2021-1G>C	-	Splicing	-	-	PVS1 PM2 PM3 PP3	P	51/ch; 52/ch	Novel
28	c.2021G>A	p.Gly674Asp	Missense	-	-	PM2 PM3 PP3 PP4	LP	57/ch	Novel
28	c.2045_2052delGTGGAGAT	p.Cys682Serfs*7	Frameshift	-	-	PVS1 PM2 PP3	P	42/ch; 45/ch	Novel
30	c.2311G>A	p.Gly771Arg	Missense	-	-	PM2 PM3 PP3 PP4	LP	92/ch	Novel
30	c.2371C>T	p.Arg791*	Nonsense	rs1060499654	P/LP	PVS1 PM2 PP3	P	51/ch; 52/ch; 64/ch; 83/H; 84/H	[8]
31	c.2407C>T	p.Gln803*	Nonsense	-	-	PVS1 PM2 PP3	P	71/H	Novel
31	c.2419G>T	p.Gly807*	Nonsense	-	-	PVS1 PM2 PP3	P	35/ch	Novel
31	c.2437_2439delC	p.Arg814Glyfs*9	Frameshift	-	-	PVS1 PM2 PP3	P	67/ch	Novel
32	c.2507G>A	p.Gly836Glu	Missense	-	-	PM2 PM3 PP3 PP4	LP	85/ch	[30]
33	c.2718delT	p.Asn908Thrfs*16	Frameshift	-	-	PVS1 PM2 PP3	P	101/ch	Novel
33	c.2724_2740delCCCAGGCACACCAGGGC	p.Asn908Lysfs*26	Frameshift	-	-	PVS1 PM2 PP3	P	66/ch	Novel
35	c.2913_2914delCA	p.Asn971Lysfs*55	Frameshift	-	-	PVS1 PM2 PP3	P	59/ch; 60/ch	Novel
36	c.2990G>A	p.Gly997Glu	Missense	rs1553762113	P/VUS	PM2 PM3 PP3 PP4	LP	53/ch; 78/ch	[31]
37	c.3071G>A	p.Gly1024Asp	Missense	-	-	PM2 PM3 PP3 PP4	LP	93/ch	Novel
37	c.3143G>A	p.Gly1048Asp	Missense	-	-	PM2 PM3 PM5 PP3 PP4	LP	90/ch	Novel
41	c.3565G>C	p.Gly1189Arg	Missense	-	-	PM2 PM3 PP3 PP4	LP	35/ch	Novel
42	c.3566G>A	p.Gly1189Glu	Missense	-	-	PM2 PM3 PP3 PP4	LP	97/ch	Novel
42	c.3575G>A	p.Gly1192Glu	Missense	-	-	PM2 PM3 PP3 PP4	LP	92/ch	Novel
42	c.3643C>T	p.Arg1215*	Nonsense	rs368434069	LP	PVS1 PM2 PP3	P	101/ch	[32]
42	c.3716G>A	p.Gly1239Glu	Missense	-	-	PM2 PM3 PP3 PP4	LP	41/ch	Novel
42	c.3725G>A	p.Gly1242Asp	Missense	-	-	PM2 PM3 PP3 PP4	LP	98/ch	[33]
Intron 43	c.3882+5G>C	-	Splicing	-	-	PS4 PM2 PM3 PP2 PP4	P	41/ch	[8]
44	c.3915_3916delCA	p.Gly1307*	Nonsense	-	-	PVS1 PM2 PP3	P	74/ch	Novel
Intron 46	c.4153+1g>a	-	Splicing	-	-	PVS1 PM2 PP3	P	99/ch	[8]
47	c.4243G > C	p.Gly1415Arg	Missense	-	-	PM2 PM3 PP3 PP4	LP	100/H	[8]
48	c.4280G > T	p.Gly1427Val	Missense	-	-	PM2 PM3 PM5 PP3 PP4	LP	94/ch; 36/ch	Novel
48	c.4318delA	p.Thr1440Profs*89	Frameshift	-	-	PVS1 PM2 PP3	P	67/ch	Novel
48	c.4378T>C	p.Cys1460Arg	Missense	-	-	PM2 PM3 PP3 PP4	LP	57/ch; 97/ch	Novel
48	c.4441C>T	p.Arg1481*	Nonsense	rs121912824	P	PVS1 PM2 PP3	P	55/ch; 69/ch	[34]
Intron 50	c.4755+1G>A	-	Splicing	-	-	PVS1 PM2 PP3	P	74/ch	Novel
51	c.4793T>G	p.Leu1598Arg	Missense	rs752452590	LP/P/VUS	PS4 PM2 PM3 PP3 PP4	P	49/ch; 91/ch; 42/ch; 53/ch; 90/ch	[9]
51	c.4825C>T	p.Arg1609*	Nonsense	rs756231749	P	PVS1 PM2 PP3	P	44/ch	[35]
52	c.4982G>A	p.Arg1661His	Missense	-	-	PM1 PM2 PM3 PP3 PP4	LP	48/ch	Novel

*P*, pathogenic; *LP*, likely pathogenic; *VUS*, variants of uncertain significance; *H*, homozygous; *ch*, compound heterozygous. The reference sequence is *COL4A3* (NM_000091.4)

### *COL4A4* variants

A total of 33 variants were identified in the *COL4A4* gene, 29 (29/33, 88%) of them were novel (Table 3). There were 14 missense variants (14/33, 42%), eight splicing variants (8/33, 24%), six frameshift variants (6/33, 18%), two synonymous variants (2/33, 6%), one nonsense variant (1/33, 3%), one exon deletion variant (1/33, 3%) and one in-frame deletion variant (1/33, 3%). Of the missense variants, seven (7/14, 50%) were glycine substitutions in the collagen domain and the other seven were missense variants located in the non-collagenous (NC) domain of α4 (IV) chain. The two synonymous variants were predicted to create an exonic splicing silencer (ESS) site and potentially altering the splicing.

**Table 3:** Characteristics of *COL4A4* variants identified in this study

Exon/intron	Nucleotide change	Amino acid change	Variation type	dbSNP reference ID	ClinVar	ACMG criteria	ACMG	Patient no./zygosity	Reference
Intron 2	c.72-26_72-23delTAAT	-	Splicing	-	-	PM2 PM3 PP3 PP4	LP	58/ch	Novel
3	c.81_86delACTCAT	p.Ile29_Leu30del	In-frame deletion	rs771943519	LP/VUS	PM2 PM3 PM4 PP3 PP4	LP	39/ch; 88/ch	[11]
Intron 3	c.114+1G>A	-	Splicing	-	-	PVS1 PM2 PP3	P	87/ch	Novel
12	c.735G>A	p.Pro245=	Coding-synonyms	-	-	PM2 PM3 PP3 PP4	LP	81/ch	Novel
Intron 12	c.735+3A>G	-	Splicing	-	-	PM2 PM3 PP3 PP4	LP	75/ch	Novel
Intron 15	c.930+1G>A	-	Splicing	-	-	PVS1 PM2 PP3	P	34/ch; 79/ch	Novel
Intron 20	c.1370-5G>T	-	Splicing	rs752509706	LB	PM2 PM3 PP3 PP4	LP	76/ch	Novel
21	c.1423G>T	p.Gly475Cys	Missense	rs1371408968	LP	PM2 PM3 PP3 PP4	LP	81/ch	Novel
25	c.1921C>T	p.Arg641*	Nonsense	-	P	PVS1 PM2 PP3 PP4	P	96/H	Novel
Intron 25	c.1987+4A>G	-	Splicing	-	-	PM2 PM3 PP3 PP4	LP	77/ch	Novel
28	c.2317delA	p.Arg773Glyfs*9	Frameshift	-	-	PVS1 PM2 PP3 PP4	P	46/ch	Novel
30	c.2678_2688delTTGGAGATGAT	p.Phe893Trpfs*66	Frameshift	-	-	PVS1 PM2 PP3 PP4	P	80/ch	Novel
30	c.2609G>T	p.Gly870Val	Missense	-	-	PM2 PM3 PM5 PP3 PP4	LP	87/ch	Novel
Intron 30	c.2717-2A>G	-	Splicing	-	-	PVS1 PM2 PP3 PP4	P	40/ch	[11]
31	c.2752G>A	p.Gly918Arg	Missense	rs372606845	-	PS4 PM2 PP3 PP4	LP	54/H	[10]
31	c.2726G>A	p.Gly909Glu	Missense	-	-	PM2 PM3 PP3 PP4	LP	75/ch	Novel
Intron 32	c.2968+1G>A	-	Splicing	-	-	PVS1 PM2 PP3 PP4	P	70/ch	Novel
33	c.3014G>A	p.Gly1005Glu	Missense	rs769138971	-	PM2 PM3 PP3 PP4	LP	40/ch	Novel
34	c.3152delG	p.Gly1051Valfs*90	Frameshift	-	-	PVS1 PM2 PP3 PP4	P	77/ch	Novel
38	c.3514G>A	p.Gly1172Arg	Missense	-	-	PM1 PM2 PP3 PP4	LP	58/ch	Novel
39	c.3636_3637delAG	p.Gly1213Argfs*39	Frameshift	-	-	PVS1 PM2 PP3 PP4	P	88/ch; 89/ch	Novel
40	c.3724G>A	p.Gly1242Ser	Missense	-	-	PM2 PM3 PP3 PP4	LP	89/ch	Novel
42	c.3990G>A	p.Pro1330=	Coding-synonyms	rs62189848	LB	PM2 PM3 PP3 PP4	LP	79/ch	Novel
46	c.4409T>C	p.Leu1470Pro	Missense	-	-	PM2 PM3 PP3 PP4	LP	39/ch	Novel
46	c.4444delC	p.Leu1482Trpfs*70	Frameshift	-	-	PVS1 PM2 PP3 PP4	P	76/ch	Novel
46	c.4363C>G	p.Pro1455Ala	Missense	-	-	PM2 PM3 PP3 PP4	LP	30/ch	Novel
47	c.4766C>T	p.Pro1589Leu	Missense	rs768974023	-	PM2 PP3 PP4	VUS	34/ch	Novel
47	c.4754_4761insC	p.Cys1588Metfs*46	Frameshift	-	-	PVS1 PM2 PP3 PP4	P	46/ch	Novel
47	c.4715C>T	p.Pro1572Leu	Missense	rs121912863	P	PS4 PM2 PM3 PP3 PP4	P	47/ch	[36]
48	c.4961C>T	p.Thr1654Met	Missense	rs771066050	-	PM2 PM3 PP3 PP4	LP	47/ch	Novel
48	Exon 48 Del	-	Exon deletion	-	-	PVS1 PM2 PP3 PP4	P	50/H	Novel
48	c.5047T>G	p.Cys1683Gly	Missense	-	-	PM2 PM3 PM5 PP3 PP4	LP	80/ch; 70/ch	Novel
48	c.4910G>A	p.Arg1637Gln	Missense	-	-	PM2 PM3 PP3 PP4	LP	30/ch	Novel

*P*, pathogenic; *LP*, likely pathogenic; *VUS*, variants of uncertain significance; *LB*, likely benign; *H*, homozygous; *ch*, compound heterozygous. The reference sequence is *COL4A4* (NM_000092.4)

### Genotype–phenotype correlations

Of the 79 patients with pathogenic variants in the *COL4A3* or *COL4A4* genes, the detailed gene variants were not available in two patients with *COL4A4* gene mutations. We divided the remaining 77 patients into three groups according to the number of missense variants. Eighteen patients had 2 missense variants, 20 patients had 1 missense variant, and 39 patients had no missense variant.

In these three groups, patients without a missense variant showed a more severe course of disease with a higher prevalence of nephrotic-range proteinuria, hearing loss and ocular abnormalities, and an earlier age compared to patients with 1 or 2 missense variants (Table 4). Patients without a missense variant progressed to CKD 5 at an earlier age (18.5 years) compared to patients with 2 missense variants at an age of 30.0 years (*p* = 0.125). Of ten patients with FSGS, five patients (50%) had no missense variant, two patients (20%) had 1 missense variant, and three patients (30%) had 2 missense variants.

**Table 4:** Clinical features of 77 ARAS patients in different groups according to the number of missense variants

	Number of missense variants			*P*
	2	1	0	
Number of patients	*n* = 18	*n* = 20	*n* = 39	
Number of patients (%) with				
Nephrotic-range proteinuria	2 (2/14, 14%)	6 (6/20, 30%)	14(14/33, 42%)	*^$^
Hearing loss	1 (1/8, 13%)	4 (4/15, 27%)	20 (20/28, 71%)	
Ocular abnormalities	0 (0/7, 0)	4(4/15, 27%)	9 (9/24, 38%)	
CKD 5	3 (3/18, 17%)	0 (0/20, 0)	5 (5/39, 13%)	
Median age (years) detected with				
Nephrotic-range proteinuria	20.0 (7.0–33.0)	18.5 (4.0–24.0)	10.0 (4.5–20.0)	*^$^
Hearing loss	33	20.0 (7.0–27.0)	10.0 (0.1–19.0)	
Ocular abnormalities	-	11.0(1.0–25.0)	11.0 (4.0–15.0)	
CKD5	30.0 (24.0–35.0)	-	18.5 (16.0–46.0)	

*p < 0.05 (0 vs. 2), ^$^*p* < 0.05 (0 vs. 1)

**Fig. 2:**
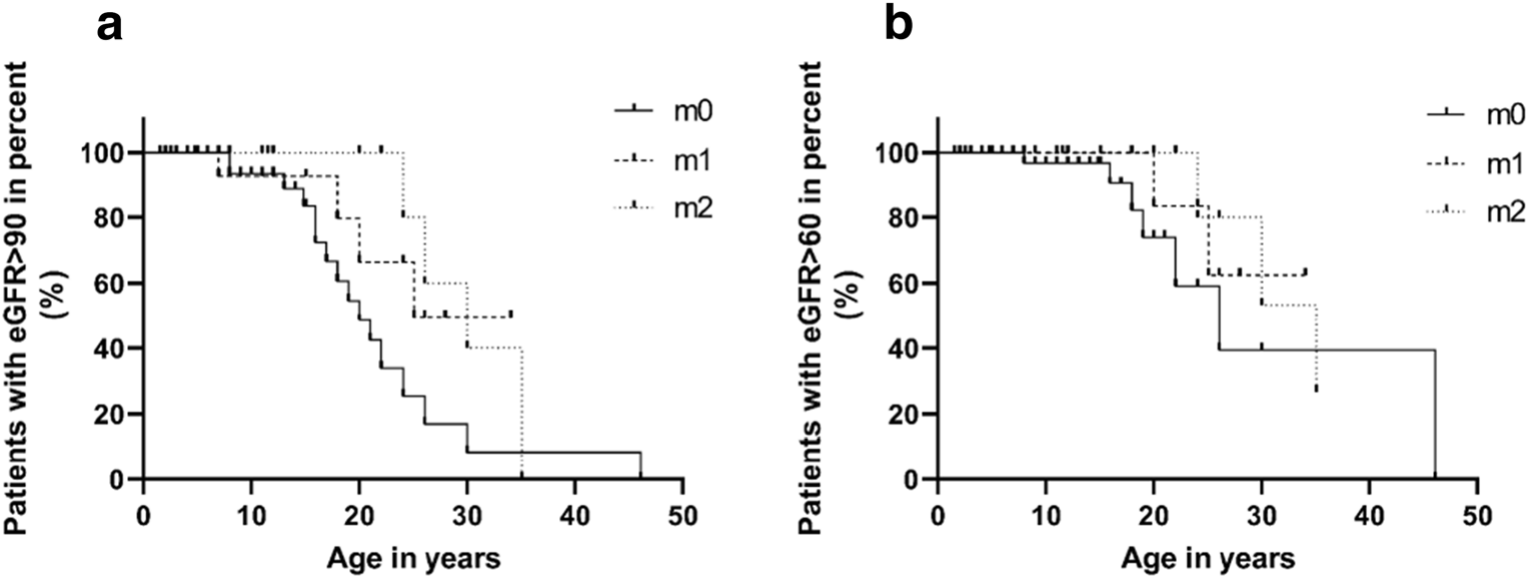
Kidney function decline occurs earlier and faster in ARAS patients with no missense variants compared with patients with one or two missense variants. **a** Patients with eGFR >90 ml/min × 1.73m^2^ in different genotypes (*p* = 0.080); **b** patients with eGFR >60 ml/min × 1.73m^2^ in different genotypes (*p* = 0.524); m0, patients with no missense variant (*n* = 39); m1, patients with one missense variant (*n* = 20); m2, patients with two missense variants (*n* = 16).

Decline of kidney function in the three groups is shown in Fig. 2. The median time with preserved kidney function (defined as eGFR >90 ml/min × 1.73m^2^, Fig. 2a; eGFR >60 ml/min × 1.73m^2^, Fig. 2b) was improved in patients with 2 missense variants compared to patients without a missense variant (30.0 vs. 20.0 years, 35.0 vs. 26.0 years, respectively) though the difference did not reach statistical significance. The probability of having a preserved kidney function (eGFR >60 ml/min × 1.73m^2^, Fig. 2b) at 25 years in ARAS patients without missense, 1 missense, and 2 missense variants was 40%, 60%, and 80%, respectively. These results demonstrate that kidney function declines earlier and faster in ARAS patients without a missense variant compared to patients with 1 or 2 missense variants.

### Nephroprotective effect of RAAS inhibition

**Fig. 3:**
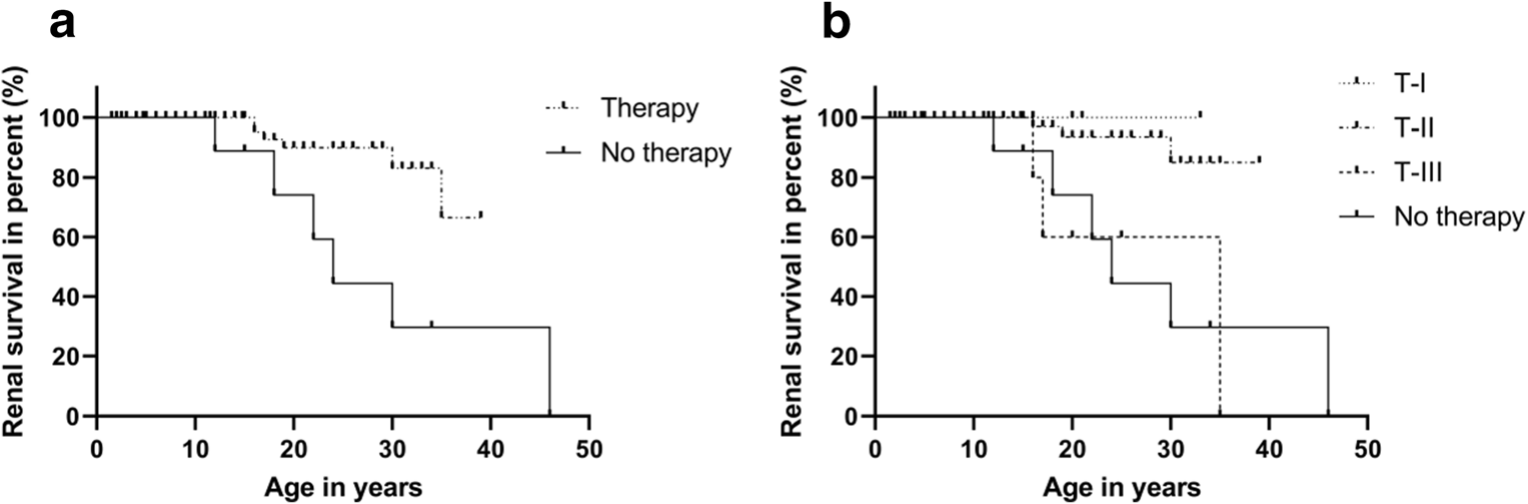
Effect of RAAS inhibition therapy on kidney survival. **a** RAAS inhibition therapy can decrease the risk of CKD 5 and delay the onset age of CKD 5 in ARAS patients (*p* = 0.004). No therapy: *n* = 22; therapy: *n* = 79. **b** RAAS inhibition therapy delayed CKD 5 in a time-dependent manner in ARAS patients (*p* = 0.002). T-I: *n* = 9; T-II: *n* = 65; T-III: *n* = 5; no. therapy: *n* = 22.

Of 101 patients, 79 patients currently received RAAS inhibition therapy (ACEi or ARB). The median age at initiation of therapy was 10 years (1.0 to 33.0 years) and the mean duration of therapy was 6.5 ± 6.0 years. There were 22 untreated patients with a median age of 11 years (2.0 to 46.0 years). Of the untreated patients, ten had only hematuria, six had CKD 5 at the beginning of diagnosis, and six were newly diagnosed as ARAS and presented with hematuria and proteinuria. In Fig. 3a, the nephroprotective effect of RAAS blockade on kidney survival (defined as starting kidney replacement therapy) is shown (*p =* 0.004). In Fig. 3b, the median kidney survival for patients without therapy was 24 years. RAAS inhibition therapy decreased the risk of CKD 5 and delayed the onset age of CKD 5 in ARAS patients depending on the time respective to the stage of disease at initiation of therapy (*p* = 0.002). In very late treated patients (T-III) with a mean duration of therapy of 9.0 years, therapy delayed onset of CKD 5 to 35 years of age (*p* = 0.949 vs. no therapy). Late treated patients (T-II) had a mean duration of therapy of 6.2 years and early treated patients (T-I) had a mean duration of therapy of 6.8 years. In both groups, T-II and T-I, the median age at onset of CKD 5 was still undefined because of their young age and the much slower progression of disease due to nephroprotective therapy (T-II vs. no therapy, *p* < 0.001; T-I vs. no therapy, *p* = 0.190; respectively).

**Fig. 4:**
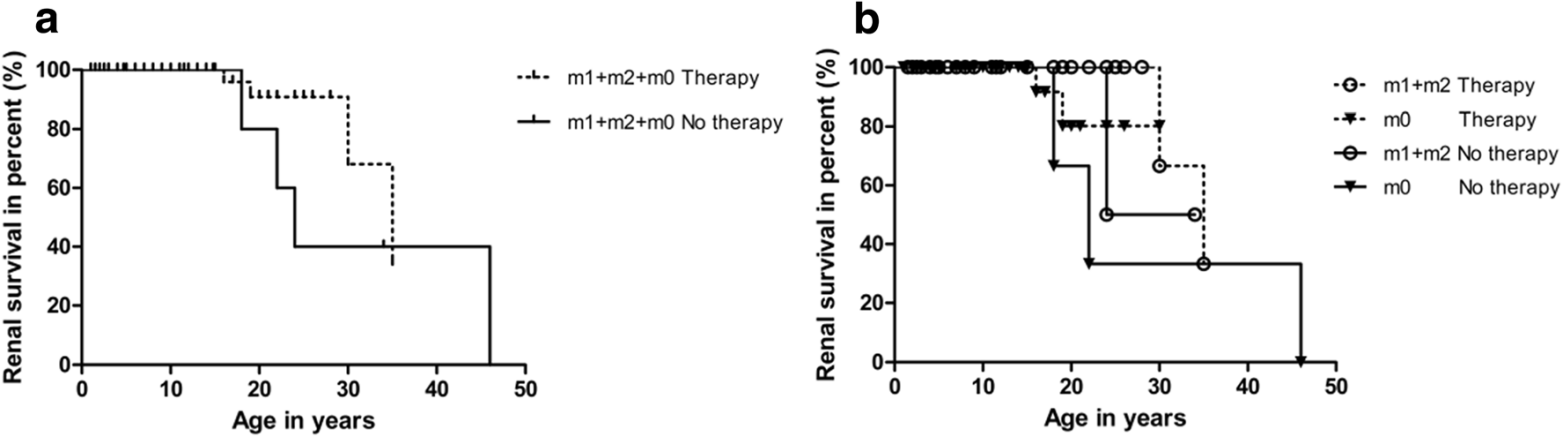
Effect of RAAS inhibition therapy on kidney survival in patients with different genotype. **a** Nephroprotective effect of RAAS inhibition on kidney survival in genetically diagnosed ARAS patients (*p* = 0.236). No therapy: *n* = 20; therapy: *n* = 57. **b** Kidney survival curve between therapy and no therapy for both patients in group m0 and group m1 + m2. m1 + m2 therapy: *n* = 28; m0 therapy: *n* = 29; m1 + m2 no therapy: *n* = 10; m0 no therapy: *n* = 10

Finally, the effect of the genotype on the response to nephroprotective therapy was analyzed in 77 patients with a genetic diagnosis (Fig. 4). The median survival time of kidney function was prolonged to 35 years in patients with RAAS inhibition therapy compared to 24 years in patients with no therapy (Fig. 4a). This result was in agreement with what we found in the paragraph above in all 101 ARAS patients with both clinical and genetic diagnosis, though the difference here did not reach statistical significance. It was found that, in spite of therapy, the onset of CKD 5 was still earlier in patients with no missense variants (m0) compared to patients with 1 or 2 missense variants (m1 + m2) (Fig. 4b). However, the difference was not statistically significant. The median age of patients with 1 or 2 missense variants (m1 + m2) was 16.5 years (range 1.5 to 35 years) in the therapy group and 7.0 years (range 2.0 to 34 years) in the no therapy group, respectively. The median age of patients with no missense variants (m0) was 11.0 years (range 2.0 to 30 years) in the therapy group and 13 years (range 1.0 to 46 years) in the no therapy group, respectively.

## Discussion

Taking advantage of the combination of the Chinese Registry Database of Hereditary Kidney Diseases and the European Alport registry, the present study describes the clinical and genetic features of 101 patients with ARAS. Furthermore, the study analyzed the genotype–phenotype correlations with a special focus on the response to nephroprotective therapy. For the first time, we confirm the nephroprotective effects of RAAS inhibition therapy in patients with ARAS.

By analyzing the available clinical data in this study, we found that hematuria was present in 100% of patients with ARAS, which was first diagnosed at a median age of 4 years. Proteinuria was diagnosed in 86% of patients at a median age of 7 years. Hearing loss was observed in 49% of patients at a median age of 10 years and ocular lesions were observed in 28% of patients at a median age of 11 years. Patients with ARAS developed CKD 5 at a median age of 20.5 years, which is in agreement with the systematic review of ARAS by Lee and coworkers, who reported CKD 5 at a median age of 21 years [17]. Of note, 39% of our patients developed nephrotic-range proteinuria at a median age of 11 years. These results suggest that ARAS should be considered in the differential diagnosis for children with hematuria and proteinuria, even for children with nephrotic-range proteinuria.

In previous reports, 10% of patients with ARAS had only diffuse thinning of the GBM [17]. In this study, all the available kidney biopsy results showed typical changes of GBM on electron microscopy, including irregular thinning and thickening, splitting, and lamellation in GBM. Thus, electronic microscopy examination of GBM in kidney biopsy remains a very reliable method for diagnosis of Alport syndrome.

One interesting aspect of our study is the opportunity to correlate the results of kidney biopsy by light microscopy with the age at biopsy. We can see that kidney pathological changes progress over time (as a spectrum from minor glomerular abnormalities resulting in an FSGS-like pattern). In other words, the older the patient with ARAS is when they receive a kidney biopsy, the more likely FSGS can become the mis-diagnosis, if one does not include electron microscopy or one does not exclude Alport syndrome as the underlying cause of FSGS. Recently, heterozygous variants in *COL4A3* were identified in FSGS patients, 12.5% of FSGS families, and one sporadic FSGS patient had heterozygous variants in the *COL4A3* gene [37]. In that report, some patients had segmental GBM thinning, but these were not classified as changes typical for Alport syndrome. Of note, FSGS is a very common finding, which can be primary or secondary [38]. It has been reported that patients with a diagnosis of TBMN and ADAS also presented FSGS [39, 40]. Fogo et al. speculated that some variants could result in an incomplete penetrance of the Alport syndrome kidney phenotype, resulting in a varying spectrum of glomerular lesions with secondary FSGS [41]. In 2018, Braunisch et al. reported one case who was erroneously considered to have hereditary FSGS at 28 years and after she developed hearing impairment at the age of 34 years, and was shown to have Alport syndrome by genetic analysis [42]. Our data very strongly support the fact that pathogenesis of the type IV collagen disease Alport syndrome leads to secondary changes, which mimic FSGS, but should not be called FSGS, because this wrong diagnosis is misleading and might trigger false and harmful immunosuppressive therapies. Thus, the new classification of Alport syndrome by “the Alport Syndrome Classification Working Group” published in 2018 can help to minimize diagnostic confusion of disorders arising from the *COL4A3* or *COL4A4* genes [43].

The present study investigated the genotype–phenotype correlation with regard to the probability and age at onset of nephrotic-range proteinuria, hearing loss, ocular abnormalities, and CKD 5 between the subgroups of patients with no. 1 and 2 missense variants. Of note, patients without a missense variant reached CKD 5 earlier, had a higher prevalence of ocular abnormalities, and had both earlier median age and higher prevalence of hearing loss and nephrotic-range proteinuria compared to patients with 1 or 2 missense variants. Moreover, eGFR declined earlier and faster in patients without a missense variant. The results indicate that the severity of disease and the speed of decline of kidney function are negatively correlated with the number of missense variants detected in ARAS. These data are in agreement with investigations in patients with XLAS, who also showed a correlation between type of variant and decline of kidney function [44].

For the first time, our study showed that in patients with ARAS, CKD 5 can be delayed by RAAS blockade in a time-dependent manner. Initiation of therapy at an earlier stage of ARAS resulted in a superior preservation of kidney function. These nephroprotective effects are in agreement with previous reports of ACEi-therapy in Alport syndrome, in which the majority of patients had XLAS [19]. Furthermore, RAAS blockade in 49 patients with different ARAS genotypes was less effective in patients without a missense variant, who had a higher prevalence and earlier age of onset of CKD 5 compared to patients with at least one missense variant. Collectively, these results demonstrate that early RAAS blockade delays onset of CKD 5 in ARAS. These data are in agreement with the investigation in patients with XLAS, who also showed a correlation between type of variant and response to nephroprotective therapy [44].

Therefore, genetic testing should be the priority choice for diagnosing Alport syndrome. It can benefit Alport patients from many aspects, such as early diagnosis, determining the hereditary modes, indicating the likely clinical course and response to nephroprotective therapy, and genetic counseling. All these advantages make genetic testing the first option instead of kidney biopsy for Alport patients. However, this does not mean genetic testing can replace kidney biopsy for all Alport patients. In some patients with suspected Alport syndrome who have no *COL4* pathogenic variants identified or gene variants of uncertain significance, GBM appearance is critical to determine the necessity of further specialized tests and diagnosis for them.

Our study has its own limitations. First, one of the inclusion criteria for patient selection was clinical diagnosis of ARAS based on typical ultrastructural changes of GBM in patients and hematuria without proteinuria or CKD 5 in both parents. This is often correct and helpful to distinguish ARAS from XLAS, but it can also be missleading in families with a coincidental kidney disease [45] or exclude some ARAS cases, as parents with a single autosomal variant are at risk of both hematuria and proteinuria [46]. Second, the low median age of the cohort and the small number of patients on kidney replacement therapy prevents us from having more robust data on CKD 5.

In conclusion, this is the largest study conducted to date in patients with ARAS, including the longest follow-up period on response to therapy. Our findings demonstrate that clinical features in patients with ARAS commonly include nephrotic-range proteinuria and FSGS in advanced stages in children and young adults. Furthermore, decline of eGFR, and age at onset of CKD 5, hearing loss, and ocular abnormalities are strongly correlated with the number of missense variants detected in ARAS. Our study provides further evidence for early use of nephroprotective RAAS blockade in young patients with ARAS.

## References

[1] Savige J, Gregory M, Gross O, Kashtan C, Ding J, Flinter F (2013). Expert guidelines for the management of Alport syndrome and thin basement membrane nephropathy. J Am Soc Nephrol.

[2] Miner JH, Baigent C, Flinter F, Gross O, Judge P, Kashtan CE, Lagas S, Savige J, Blatt D, Ding J, Gale DP, Midgley JP, Povey S, Prunotto M, Renault D, Skelding J, Turner AN, Gear S (2014). The 2014 International Workshop on Alport Syndrome. Kidney Int.

[3] Zhang X, Zhang Y, Zhang Y, Gu H, Chen Z, Ren L, Lu X, Chen L, Wang F, Liu Y, Ding J (2018). X-linked Alport syndrome: pathogenic variant features and further auditory genotype-phenotype correlations in males. Orphanet J Rare Dis.

[4] Savige J, Sheth S, Leys A, Nicholson A, Mack HG, Colville D (2015). Ocular features in Alport syndrome: pathogenesis and clinical significance. Clin J Am Soc Nephrol.

[5] Zhang Y, Ding J (2018). Renal, auricular, and ocular outcomes of Alport syndrome and their current management. Pediatr Nephrol.

[6] Wang F, Zhao D, Ding J, Zhang H, Zhang Y, Yu L, Xiao H, Yao Y, Zhong X, Wang S (2012). Skin biopsy is a practical approach for the clinical diagnosis and molecular genetic analysis of X-linked Alport’s syndrome. J Mol Diagn.

[7] Storey H, Savige J, Sivakumar V, Abbs S, Flinter FA (2013). COL4A3/COL4A4 mutations and features in individuals with autosomal recessive Alport syndrome. J Am Soc Nephrol.

[8] Zhang Y, Wang F, Ding J, Zhang H, Zhao D, Yu L, Xiao H, Yao Y, Zhong X, Wang S (2012). Genotype-phenotype correlations in 17 Chinese patients with autosomal recessive Alport syndrome. Am J Med Genet A.

[9] Oka M, Nozu K, Kaito H, Fu XJ, Nakanishi K, Hashimura Y, Morisada N, Yan K, Matsuo M, Yoshikawa N, Vorechovsky I, Iijima K (2014). Natural history of genetically proven autosomal recessive Alport syndrome. Pediatr Nephrol.

[10] Fallerini C, Dosa L, Tita R, Del Prete D, Feriozzi S, Gai G, Clementi M, La Manna A, Miglietti N, Mancini R, Mandrile G, Ghiggeri GM, Piaggio G, Brancati F, Diano L, Frate E, Pinciaroli AR, Giani M, Castorina P, Bresin E, Giachino D, De Marchi M, Mari F, Bruttini M, Renieri A, Ariani F (2014). Unbiased next generation sequencing analysis confirms the existence of autosomal dominant Alport syndrome in a relevant fraction of cases. Clin Genet.

[11] Morinière V, Dahan K, Hilbert P, Lison M, Lebbah S, Topa A, Bole-Feysot C, Pruvost S, Nitschke P, Plaisier E, Knebelmann B, Macher MA, Noel LH, Gubler MC, Antignac C, Heidet L (2014). Improving mutation screening in familial hematuric nephropathies through next generation sequencing. J Am Soc Nephrol.

[12] Fallerini C, Baldassarri M, Trevisson E, Morbidoni V, La Manna A, Lazzarin R, Pasini A, Barbano G, Pinciaroli AR, Garosi G, Frullanti E, Pinto AM, Mencarelli MA, Mari F, Renieri A, Ariani F (2017). Alport syndrome: impact of digenic inheritance in patients management. Clin Genet.

[13] Zhang Y, Ding J, Zhang H, Yao Y, Xiao H, Wang S, Wang F (2019). Effect of heterozygous pathogenic COL4A3 or COL4A4 variants on patients with X-linked Alport syndrome. Mol Genet Genomic Med.

[14] Jais JP, Knebelmann B, Giatras I, De Marchi M, Rizzoni G, Renieri A, Weber M, Gross O, Netzer KO, Flinter F, Pirson Y, Verellen C, Wieslander J, Persson U, Tryggvason K, Martin P, Hertz JM, Schröder C, Sanak M, Krejcova S, Carvalho MF, Saus J, Antignac C, Smeets H, Gubler MC (2000). X-linked Alport syndrome: natural history in 195 families and genotype- phenotype correlations in males. J Am Soc Nephrol.

[15] Gross O, Netzer KO, Lambrecht R, Seibold S, Weber M (2002). Meta-analysis of genotype-phenotype correlation in X-linked Alport syndrome: impact on clinical counselling. Nephrol Dial Transplant.

[16] Horinouchi T, Nozu K, Yamamura T, Minamikawa S, Omori T, Nakanishi K, Fujimura J, Ashida A, Kitamura M, Kawano M, Shimabukuro W, Kitabayashi C, Imafuku A, Tamagaki K, Kamei K, Okamoto K, Fujinaga S, Oka M, Igarashi T, Miyazono A, Sawanobori E, Fujimaru R, Nakanishi K, Shima Y, Matsuo M, Ye MJ, Nozu Y, Morisada N, Kaito H, Iijima K (2018). Detection of splicing abnormalities and genotype-phenotype correlation in X-linked Alport syndrome. J Am Soc Nephrol.

[17] Lee JM, Nozu K, Choi DE, Kang HG, Ha IS, Cheong HI (2019). Features of autosomal recessive Alport syndrome: a systematic review. J Clin Med.

[18] Gross O, Beirowski B, Koepke ML, Kuck J, Reiner M, Addicks K, Smyth N, Schulze-Lohoff E, Weber M (2003). Preemptive ramipril therapy delays renal failure and reduces renal fibrosis in COL4A3-knockout mice with Alport syndrome. Kidney Int.

[19] Gross O, Licht C, Anders HJ, Hoppe B, Beck B, Tönshoff B, Höcker B, Wygoda S, Ehrich JH, Pape L, Konrad M, Rascher W, Dötsch J, Müller-Wiefel DE, Hoyer P, Knebelmann B, Pirson Y, Grunfeld JP, Niaudet P, Cochat P, Heidet L, Lebbah S, Torra R, Friede T, Lange K, Müller GA, Weber M, Study Group Members of the Gesellschaft für Pädiatrische Nephrologie (2012). Early angiotensin-converting enzyme inhibition in Alport syndrome delays renal failure and improves life expectancy. Kidney Int.

[20] Kashtan CE, Ding J, Gregory M, Gross O, Heidet L, Knebelmann B, Rheault M, Licht C, Alport Syndrome Research Collaborative (2013). Clinical practice recommendations for the treatment of Alport syndrome: a statement of the Alport Syndrome Research Collaborative. Pediatr Nephrol.

[21] Zhang Y, Wang F, Ding J, Zhang H, Liu X, Wang S, Xiao H, Yao Y, Liu J, Zhong X, Guan N, Su B, Wu G, Yu L (2016). Long-term treatment by ACE inhibitors and angiotensin receptor blockers in children with Alport syndrome. Pediatr Nephrol.

[22] Gross O, Kashtan CE, Rheault MN, Flinter F, Savige J, Miner JH, Torra R, Ars E, Deltas C, Savva I, Perin L, Renieri A, Ariani F, Mari F, Baigent C, Judge P, Knebelman B, Heidet L, Lagas S, Blatt D, Ding J, Zhang Y, Gale DP, Prunotto M, Xue Y, Schachter AD, Morton LCG, Blem J, Huang M, Liu S, Vallee S, Renault D, Schifter J, Skelding J, Gear S, Friede T, Turner AN, Lennon R (2017). Advances and unmet needs in genetic, basic and clinical science in Alport syndrome: report from the 2015 International Workshop on Alport Syndrome. Nephrol Dial Transplant.

[23] Savige J, Ariani F, Mari F, Bruttini M, Renieri A, Gross O, Deltas C, Flinter F, Ding J, Gale DP, Nagel M, Yau M, Shagam L, Torra R, Ars E, Hoefele J, Garosi G, Storey H (2019). Expert consensus guidelines for the genetic diagnosis of Alport syndrome. Pediatr Nephrol.

[24] Richards S, Aziz N, Bale S, Bick D, Das S, Gastier-Foster J, Grody WW, Hegde M, Lyon E, Spector E, Voelkerding K, Rehm HL, ACMG Laboratory Quality Assurance Committee (2015). Standards and guidelines for the interpretation of sequence variants: a joint consensus recommendation of the American College of Medical Genetics and Genomics and the Association for Molecular Pathology. Genet Med.

[25] Rana K, Tonna S, Wang YY, Sin L, Lin T, Shaw E, Mookerjee I, Savige J (2007). Nine novel COL4A3 and COL4A4 mutations and polymorphisms identified in inherited membrane diseases. Pediatr Nephrol.

[26] Webb BD, Brandt T, Liu L, Jalas C, Liao J, Fedick A, Linderman MD, Diaz GA, Kornreich R, Trachtman H, Mehta L, Edelmann L (2014). A founder mutation in COL4A3 causes autosomal recessive Alport syndrome in the Ashkenazi Jewish population. Clin Genet.

[27] Miyagawa M, Naito T, Nishio SY, Kamatani N, Usami S (2013). Targeted exon sequencing successfully discovers rare causative genes and clarifies the molecular epidemiology of Japanese deafness patients. PLoS One.

[28] Heidet L, Arrondel C, Forestier L, Cohen-Solal L, Mollet G, Gutierrez B, Stavrou C, Gubler MC, Antignac C (2001). Structure of the human type IV collagen gene COL4A3 and mutations in autosomal Alport syndrome. J Am Soc Nephrol.

[29] Savige J, Storey H, Il Cheong H, Gyung Kang H, Park E, Hilbert P, Persikov A, Torres-Fernandez C, Ars E, Torra R, Hertz JM, Thomassen M, Shagam L, Wang D, Wang Y, Flinter F, Nagel M (2016). X-linked and autosomal recessive Alport syndrome: pathogenic variant features and further genotype-phenotype correlations. PLoS One.

[30] Zhang H, Ding J, Wang F, Zhao D (2011). Mutation detection of COL4An gene based on mRNA of peripheral blood lymphocytes and prenatal diagnosis of Alport syndrome in China. Nephrology.

[31] Liu JH, Wei XX, Li A, Cui YX, Xia XY, Qin WS, Zhang MC, Gao EZ, Sun J, Gao CL, Liu FX, Wu QY, Li WW, Asan LZH, Li XJ (2017). Novel mutations in COL4A3, COL4A4, and COL4A5 in Chinese patients with Alport syndrome. PLoS One.

[32] Tazón Vega B, Badenas C, Ars E, Lens X, Milà M, Darnell A, Torra R (2003). Autosomal recessive Alport’s syndrome and benign familial hematuria are collagen type IV diseases. Am J Kidney Dis.

[33] Hou P, Chen Y, Ding J, Li G, Zhang H (2007). A novel mutation of COL4A3 presents a different contribution to Alport syndrome and thin basement membrane nephropathy. Am J Nephrol.

[34] Lemmink HH, Mochizuki T, van den Heuvel LP, Schröder CH, Barrientos A, Monnens LA, van Oost BA, Brunner HG, Reeders ST, Smeets HJ (1994). Mutations in the type IV collagen alpha 3 (COL4A3) gene in autosomal recessive Alport syndrome. Hum Mol Genet.

[35] Warejko JK, Tan W, Daga A, Schapiro D, Lawson JA, Shril S, Lovric S, Ashraf S, Rao J, Hermle T, Jobst-Schwan T, Widmeier E, Majmundar AJ, Schneider R, Gee HY, Schmidt JM, Vivante A, van der Ven AT, Ityel H, Chen J, Sadowski CE, Kohl S, Pabst WL, Nakayama M, Somers MJG, Rodig NM, Daouk G, Baum M, Stein DR, Ferguson MA, Traum AZ, Soliman NA, Kari JA, El Desoky S, Fathy H, Zenker M, Bakkaloglu SA, Müller D, Noyan A, Ozaltin F, Cadnapaphornchai MA, Hashmi S, Hopcian J, Kopp JB, Benador N, Bockenhauer D, Bogdanovic R, Stajić N, Chernin G, Ettenger R, Fehrenbach H, Kemper M, Munarriz RL, Podracka L, Büscher R, Serdaroglu E, Tasic V, Mane S, Lifton RP, Braun DA, Hildebrandt F (2018). Whole exome sequencing of patients with steroid-resistant nephrotic syndrome. Clin J Am Soc Nephrol.

[36] Boye E, Mollet G, Forestier L, Cohen-Solal L, Heidet L, Cochat P, Grünfeld JP, Palcoux JB, Gubler MC, Antignac C (1998). Determination of the genomic structure of the COL4A4 gene and of novel mutations causing autosomal recessive Alport syndrome. Am J Hum Genet.

[37] Xie J, Wu X, Ren H, Wang W, Wang Z, Pan X, Hao X, Tong J, Ma J, Ye Z, Meng G, Zhu Y, Kiryluk K, Kong X, Hu L, Chen N (2014). COL4A3 mutations cause focal segmental glomerulosclerosis. J Mol Cell Biol.

[38] Meyrier A (2005). Mechanisms of disease: focal segmental glomerulosclerosis. Nat Clin Pract Nephrol.

[39] Voskarides K, Damianou L, Neocleous V, Zouvani I, Christodoulidou S, Hadjiconstantinou V, Ioannou K, Athanasiou Y, Patsias C, Alexopoulos E, Pierides A, Kyriacou K, Deltas C (2007). COL4A3/COL4A4 mutations producing focal segmental glomerulosclerosis and renal failure in thin basement membrane nephropathy. J Am Soc Nephrol.

[40] Gast C, Pengelly RJ, Lyon M, Bunyan DJ, Seaby EG, Graham N, Venkat-Raman G, Ennis S (2016). Collagen (COL4A) mutations are the most frequent mutations underlying adult focal segmental glomerulosclerosis. Nephrol Dial Transplant.

[41] Vischini G, Kapp ME, Wheeler FC, Hopp L, Fogo AB (2018). A unique evolution of the kidney phenotype in a patient with autosomal recessive Alport syndrome. Hum Pathol.

[42] Braunisch MC, Büttner-Herold M, Günthner R, Satanovskij R, Riedhammer KM, Herr PM, Klein HG, Wahl D, Küchle C, Renders L, Heemann U, Schmaderer C, Hoefele J (2018). Heterozygous COL4A3 variants in histologically diagnosed focal segmental glomerulosclerosis. Front Pediatr.

[43] Kashtan CE, Ding J, Garosi G, Heidet L, Massella L, Nakanishi K, Nozu K, Renieri A, Rheault M, Wang F, Gross O (2018). Alport syndrome: a unified classification of genetic disorders of collagen IV α345: a position paper of the Alport Syndrome Classification Working Group. Kidney Int.

[44] Yamamura T, Horinouchi T, Nagano C, Omori T, Sakakibara N, Aoto Y, Ishiko S, Nakanishi K, Shima Y, Nagase H, Takeda H, Rossanti R, Ye MJ, Nozu Y, Ishimori S, Ninchoji T, Kaito H, Morisada N, Iijima K, Nozu K (2020). Genotype-phenotype correlations influence the response to angiotensin-targeting drugs in Japanese patients with male X-linked Alport syndrome. Kidney Int.

[45] Savige J (2020). Alport syndrome: deducing the mode of inheritance from the presence of haematuria in family members. Pediatr Nephrol.

[46] Temme J, Peters F, Lange K, Pirson Y, Heidet L, Torra R, Grunfeld JP, Weber M, Licht C, Müller GA, Gross O (2012). Incidence of renal failure and nephroprotection by RAAS inhibition in heterozygous carriers of X-chromosomal and autosomal recessive Alport mutations. Kidney Int.

